# Snail Inventories for Short‐Term Impacts: Methodological Differences and Their Underlying Causes

**DOI:** 10.1002/ece3.74518

**Published:** 2026-10-10

**Authors:** Nóra Kriston‐Kőműves, Roland Farkas, Zoltán Barta

**Affiliations:** ^1^ HUN‐REN‐DE Behavioural Ecology Research Group, Department of Evolutionary Zoology and Human Biology University of Debrecen Debrecen Hungary; ^2^ Bükk National Park Directorate Eger Hungary

**Keywords:** land snails survey, snail shell chemical resistance, snail shell physical resistance, species overrepresentation, survey bias

## Abstract

In research on terrestrial gastropod communities, a fundamental methodological question is whether to include empty shells in the investigations alongside living individuals. In this study, we examined potential biases introduced in the analyses by the inclusion of fresh empty shells (with intact periostracum). We started by reanalysing a dataset about the short‐term effects of mowing and mulching on a grassland snail community. For the original analysis, only live specimen data were used, whereas here we used a so‐called combined dataset including live individuals and fresh empty shells. While the live data revealed significant negative impacts of mowing on the snail community, these effects were undetectable with the combined data. Concurrently, we observed substantial interspecific variation in the overrepresentation value among species (the ratio of a species' combined abundance to its live abundance), which represents a distortion in the distribution of the total number of individuals across species. To explore the underlying causes of this phenomenon, we investigated the physical and chemical resistance of the shells of eleven species under laboratory conditions and simulated the effects of life history traits and shell‐degradation rate on overrepresentation using a multistage population model. Contrary to expectations, our results indicated that this variation in overrepresentation was not explained by shell resistance but rather by shell size: significantly higher overrepresentation was observed in smaller species, even though their shells are weaker, so may decompose faster. This relationship may be driven by the faster life cycles and higher turnover rates of smaller species, a finding corroborated by our population model. We concluded that the inclusion of empty shells can significantly distort the perception of the instantaneous state of the community and mask short‐term temporal changes. We recommend prioritising live data for detecting short‐term impacts and explicitly stating and justifying the chosen data type in scientific publications.

## Graphical abstract

This study investigates the effects of including fresh empty shells in terrestrial gastropod community surveys and evaluates the resulting bias by combining field data, laboratory experiments and population modelling. We found that incorporating fresh empty shells can mask short‐term changes in community composition and introduce species‐specific biases that are primarily associated with shell size rather than shell resistance. Our results highlight the importance of prioritising live individuals when assessing short‐term ecological responses and of clearly reporting the type of data used in snail community surveys.

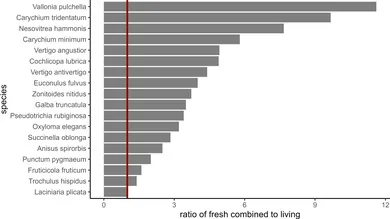

## 1 Introduction

Animal remains are widely utilised in ecological studies, especially in those based on observational evaluation and genetics. These materials can provide a record of species occurrence and abundance that might be difficult to capture through direct observation of living individuals. In the case of terrestrial vertebrates, faeces, bones and shed integumentary parts (feathers, fur, skin) are widely used (Brom 1986; Chame 2003; Solberg et al. 2006). However, the prerequisite for these unique sampling methods is a thorough knowledge of the given taxon and a deep understanding of how the animal material could have changed after being left (Henderson 2021).

Terrestrial snails (excluding slugs) possess an external calcareous shell that can persist for a long time after an individual's demise. These hard shells provide an excellent opportunity to identify individuals even after their death (Welter‐Schultes 2012). This prolonged degradation, however, means that, at any given time, both living snails and the remains of those long deceased can coexist within the same habitat. As terrestrial snails are recognised as sensitive indicators of environmental change and are frequently used as bioindicators (Pech et al. 2015; Wehner et al. 2019), it is especially important to clarify how remnants of dead snails (i.e., their shells) can influence their ecological studies, when researchers are interested in the number of individuals (Liew et al. 2008; Cernohorsky et al. 2010; Schilthuizen 2011; Farkas et al. 2025).

The most common malacological sampling method in terrestrial habitats involves collecting and processing leaf litter. During such sampling, all shells present on a defined area are collected, irrespective of whether they belong to living individuals or represent post‐mortem remains. Subsequently, researchers must decide whether to include only live individuals or also incorporate empty shells into their analyses (Liew et al. 2008; Cernohorsky et al. 2010; Schilthuizen 2011; Altaf et al. 2017).

Traditionally, empty shells are used in studies of terrestrial snail communities. Researchers often differentiate between fresh and old shells based on quality (e.g., presence of intact periostracum in fresh shells) and then analyse either the former group or both. However, a critical methodological oversight often occurs: presumably because of its time‐consuming nature, fresh but empty shells (i.e., remnants of dead individuals) are rarely separated from living ones (i.e., still containing the body), resulting in a ‘combined’ dataset of living individuals and remnants of presumably recently died ones (Altaf et al. 2017).

Considering empty shells as individuals present for community analyses, however, carries inherent risks. First, the abundance of species will certainly be inflated. Second, dead individuals represented only by their shells at sampling obviously no longer participate in population processes. Consequently, estimates of population and community parameters based on combined datasets can be severely distorted, which, for instance, can limit the value of experimental evaluation of conservation measures. The problem can be exacerbated by the possibility that the empty shells of different species can erode at different rates, i.e., they can be present in the environment for different lengths of time, meaning that the biases introduced by counting empty shells can vary across species (Sólymos et al. 2009; Cernohorsky et al. 2010).

The persistence of empty shells, and thus the potential for bias, can be influenced by numerous factors. The duration of shell decomposition can be affected by habitat characteristics (Wäreborn 1969; Menez 2002; Rundell and Cowie 2004; Cernohorsky et al. 2010; Albano 2014), shell structure and size (Menez 2002; Pearce 2008; Říhová et al. 2018). This highlights that empty shell assemblages represent time‐averaged samples over longer temporal scales, differing from the snapshot of a living community (Kidwell and Tomasovych 2013).

Nevertheless, the potential benefits of including empty shells should also be considered. Over longer time scales, such as when comparing communities across decades, the combined data type is more likely to capture the full species pool present during a given period (Pearce 2008). Whenever differences in relative species abundances within the community are not a primary concern, there is no strong rationale for excluding empty shells from the dataset. Furthermore, when populations are to be characterised using DNA‐based methods rather than direct observation of individuals, empty shells may provide a valuable non‐invasive source of material (Efstratiou et al. 2025).

Despite the acknowledgement of these influencing factors, experimental investigations on how they systematically affect the outcomes of short‐term impact studies are notably lacking (Liew et al. 2008; Sólymos et al. 2009; Schilthuizen 2011). In ecological research, while biases cannot always be eliminated due to the biology of the study organism or resource limitations (Schilthuizen 2011), it is crucial to strive for methods that track ecological processes as accurately as possible (Elphick 2008). The decision to include or exclude empty shells and define the criteria for doing so should therefore be a mindful process, detailed, justified and published within any study (Cernohorsky et al. 2010).

While two decades ago these potential biases were already mentioned in the literature, their practical consideration in snail ecological research is still not evident. Only rarely have researchers explicitly examined whether the inclusion of empty shells influences the outcomes of studies investigating the effects of environmental processes or interventions. For instance, Hylander et al. (2004) found that clear‐cutting in boreal forests reduced snail species richness and abundance, and their results were not affected by whether analyses were conducted using only living individuals or a combination of living individuals and empty shells. In contrast, Boschi and Baur (2008) noted that the presence of species persisting only as empty shells could have influenced their results in a study of grazed grasslands. Including empty shells also affected the comparison of the malacofauna of fens with different mineral content (Cernohorsky et al. 2010).

In this study, we employed a comparative experimental approach to evaluate the efficacy and potential biases associated with different snail inventory methods. Specifically, we investigated how the inclusion of empty shells influences the assessment of snail communities in the context of short‐term ecological impacts.

To address this question, we re‐analysed data collected for a short‐term impact study on snail communities, originally investigating the effects of grassland management (Farkas et al. 2025). In the original analyses, only live individuals were considered, but data on freshly dead shells were also collected. Here, we re‐run the original impact assessment analyses using combined data (live and freshly dead individuals together) and compare the results of the two analyses.

The accumulation rate of empty shells is primarily determined by two key processes: degradation and production. In this context, degradation depends on the resistance of the shells to environmental factors, while production is governed by the fecundity and mortality of individuals. To understand the former, we conducted laboratory experiments to assess the physical and chemical resistance of the shells and how these resistance measures might correlate with the biases caused by the empty shells. Here, physical resistance refers to what is commonly termed ‘crushing resistance’ in the literature, i.e., the minimum external load required to crush a shell (Johnson 2021). The shells of terrestrial gastropods occurring close to the soil surface are frequently exposed to compressive forces and physical impacts, which may cause them to break completely and thus become no longer detectable, or to sustain damage that excludes them from the fresh empty shell category (e.g., cracking or abrasion). In our study, chemical resistance refers to resistance to acidic solutions. In particular, the dissolution of calcium carbonate may reduce shell strength and integrity. In the natural environment, empty shells may be exposed to organic acids exuded by plant roots representing a relatively continuous source of chemical stress in the upper soil layer, whereas potentially acidic precipitation represents a more intermittent source of exposure. If shells of different species differ in their resistance to these environmental factors, this may affect their persistence and decomposition rates (Pearce 2008). Consequently, empty shells may remain in the population for different lengths of time after the death of the individual, potentially resulting in species‐specific differences in the representation of empty shells in datasets combining live individuals and fresh empty shells. We also constructed a structured population model, including the parameters of shell production, to clarify observed relationships (Caswell 2006).

## 2 Materials and Methods

### 2.1 The Original Data

The original research, conducted by Farkas et al. (2025), aimed to evaluate the short‐term impact of mulching and mowing, as grassland management methods, on snail communities. Shortly, two study sites were established in two wet meadows (Western Bükk region, Northern Hungary, Europe). These areas have been withdrawn from agricultural use for decades, free from mowing, grazing, or other management practices. Both areas represent flat and horizontal landscape sections devoid of trees and shrubs and are characterised by tall sedge vegetation. Their dominant species is the swamp sedge ( *Carex acutiformis* ), with characteristic species including common loosestrife ( *Lysimachia vulgaris* ), purple loosestrife ( *Lythrum salicaria* ), cabbage thistle (*Cirsium oleraceum*) and stinging nettle ( *Urtica dioica* ).

In both meadows an identical experimental design was applied for both treatments and sampling. In both, a 45 × 15 metre area was designated, and these areas were divided into nine 5 × 15 metre plots, of which three received control, three mowing, and three mulching treatments, arranged randomly. To ensure a nearly even distribution of sampling points, each plot was further divided into five 5 × 3 metre sampling units.

Data from two sampling periods were included in the analysis. The first sampling occurred at the end of June 2021, a few days before the single treatment application, and the second a little more than a year later, in mid‐August 2022. The precise dates were selected to ensure comparable weather conditions across both sites and sampling occasions. One sample was collected per sampling unit, comprising leaf litter, vegetation and the uppermost 1 cm of the soil layer within a 25 × 25 centimetre quadrat. These samples were stored in airtight plastic bags in a cool and dark place. The next day, gastropod shells were extracted using a flotation method and subsequently preserved in 70% ethanol. Slugs were not included in the study.

Species‐level identification was performed by one of us (RF), following the taxonomic keys of Welter‐Schultes (2012), Kerney et al. (1983) and Glöer and Meier‐Brook (2003). The contents of each sample were recorded individually, distinguishing between old shells, fresh empty shells and live individuals. We identified these conditions in the most common way: shells with a body inside were classified as live individuals, and empty shells with intact periostracum as fresh ones. Empty shells without intact periostracum were considered old shells and were not part of our analysis. Given RF's decades‐long experience in snail shells identification, we consider that it is unlikely that misidentification of either the species or the types of shells can cause significant bias in our dataset. Therefore, this dataset provided an excellent opportunity to compare the two data types: live (only living individuals) and combined (live individuals and fresh empty shells).

The datasets used in this study are available in the associated repository (Kriston‐Kőműves et al. 2026).

### 2.2 Comparing Live and Combined Data

In the original study (Farkas et al. 2025), only data from live individuals were analysed and data from the two study sites were processed together. Initially, we replicated this analysis using combined data, which encompassed the following procedures.

Generalised Linear Mixed Models (GLMM) were fitted to total abundance, species richness and Pielou's evenness index (Pielou 1966) to examine the effects of sampling occasion (pre‐ or post‐treatment), management methods, and their interaction. Within this framework, the impact of specific management methods is indicated by a significant interaction between sampling occasion and treatment type. All analyses were conducted in the R statistical environment (v4.5.1, R Core Team 2025). Model fitting was performed using the ‘glmmTMB’ R package (v1.1.7, Brooks et al. 2017), while model diagnostics and fit evaluation were conducted with the ‘DHARMa’ R package (v0.4.6, Hartig 2022). Post hoc comparisons and group effects were assessed using the ‘emmeans’ R package (v1.8.4–1, Lenth 2023). For abundance data, we used a negative binomial error distribution, whereas normal error distributions were used for species richness and Pielou's index. The random effect structure included the unique identifiers of sampling units nested within plot IDs, which were further nested within site IDs for the abundance and species richness models. In the case of Pielou's evenness index, the random factors explained such a negligible proportion of the variance that the model failed to converge; consequently, random factors were omitted from this specific model.

We also investigated how species differ in the level of bias caused by including freshly died individuals in the analyses. First, an overrepresentation value was calculated for each species (combined abundance of the species divided by abundance of live individuals of the species) based on the pre‐treatment survey data. After that, we fitted models to investigate how size, shape and resistance variables (see below) influence overrepresentation. The metric of overrepresentation presents analytical challenges due to its distributional properties, specifically its lower bound of one. To address this, we adopted an alternative approach by entering the counts of live (as success) and freshly died (as failure) into the analyses using generalised linear models with a binomial error distribution. The significance of the models was assessed using Wald *z*‐tests, *t*‐tests and likelihood ratio *χ* ^2^ tests.

### 2.3 Resistance and Proportions of Shells

Laboratory degradation experiments were conducted to compare the physical and chemical resistance of the shells across different species. For this purpose, we utilised specimens collected directly during the original research. These individuals were live‐collected and stored in ethanol (70%). Only adult individuals were examined, and we excluded any damaged or visibly worn (e.g., faded or heavily scratched) shells.

Individuals of every acquired species were dried at room temperature and assigned unique IDs. Top‐view photographs were taken of the shells in a recumbent position: i.e., the position that the shell naturally assumes when placed on a flat surface, which depends on its shape (300 dpi, Dino Lite digital microscope, DinoCapture 2.0, Dino‐Lite Europe, Jupiter Tech Kft.; Huawei P30 Pro). From these images, the maximum diameter (hereafter length) and the width (the segment perpendicular to the midpoint of the length) were measured (ImageJ). We used these measures to calculate the size (length * width) and the shape (width/length) of the shells. Species with elongated shells have a lower shape value, while those with flat and round shells have a higher, close to 1 value.

Eleven species were included in the resistance analysis because only these provided sufficient sample sizes. Ten individuals per species were allocated for physical resistance testing, and ten for chemical resistance testing.

Physical resistance was defined as the weight at which the shell fractured. We placed the shell in a recumbent position on a flat, hard plastic surface (inside of a Petri dish). To ensure an even distribution of the load, another plastic Petri dish was placed over the shell, followed by a lightweight container (Figure 1). Water was then added gradually to the container until the fracture point was reached. The weight was measured using an analytical balance and rounded to the nearest tenth of a gram. These values were subsequently averaged for each species. Physical resistance was analysed with linear models with a Gaussian error distribution.

**FIGURE 1:**
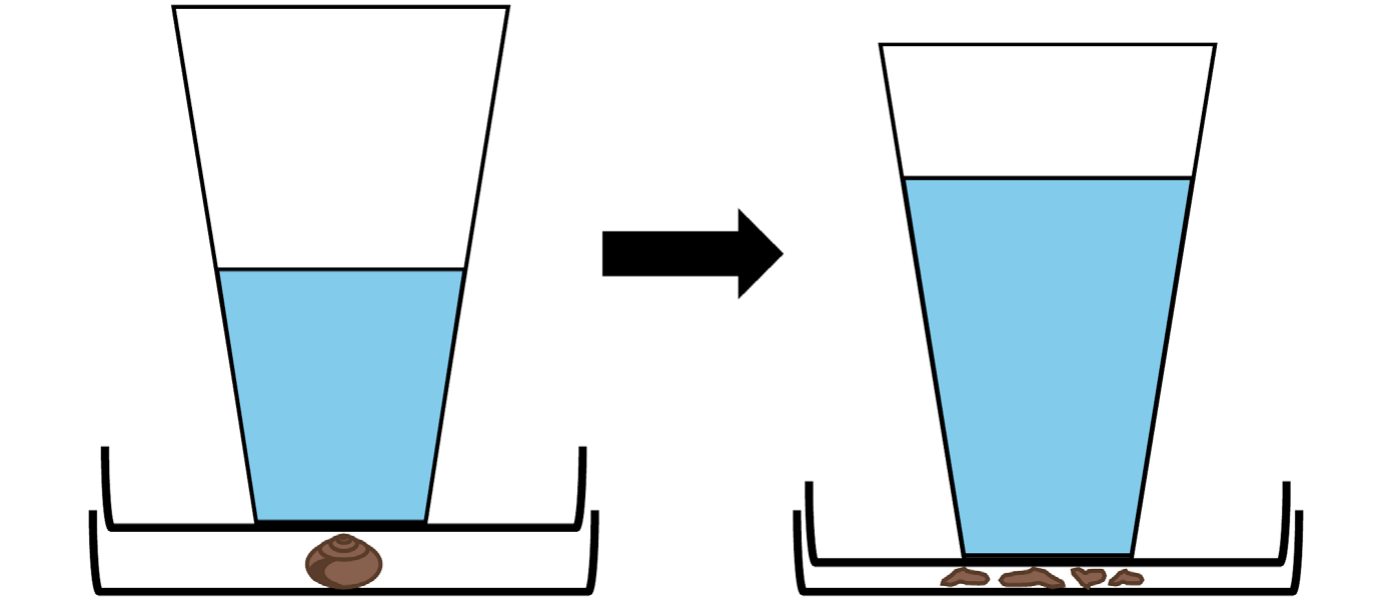
Illustration of the physical resistance measurement. The shell is between two petri dishes in the middle. The plastic cup was gradually filled with water until the shell broke.

For the chemical resistance assessment, specimens were immersed in 50% (v/v) acetic acid solution for 15 min, using 12 mL of solution per individual. During this period, some shells lost their shape due to the dissolution of calcium carbonate (dissolved shells), while others kept it. Based on these observations, a dissolution rate was calculated as the number of dissolved shells divided by the total number of shells for each species. Note: this measure is the inverse of chemical resistance, i.e., higher values indicate lower resistance. Factors influencing chemical resistance were analysed in generalised linear models with betabinomial error distribution.

### 2.4 Production of Shells

Based on the literature, smaller Gastropods may produce a higher number of shells due to their fast pace of life (i.e., quicker reproduction and higher mortality rate; Künkel 1928; Frömmig 1954; Uminski and Focht 1979; Baur 1989; Myzyk 2011; Maltz and Sulikowska‐Drozd 2012; Welter‐Schultes 2012). As this might affect the number of shells of dead individuals, we developed a Leslie matrix model of a structured snail population (Caswell 2006). We assumed no migration. Three stages were modelled. (1) Young snails: they were born between the current and previous censuses, and they cannot reproduce, (2) adult individuals: they are fully grown and able to reproduce; young become adults by the time of the next census; they can live for several censuses, (3) dead shells: they are the fresh remnant of individuals who died before the census; they can have died several censuses ago. The life cycle diagram and the transition parameters are given in Figure 2.

**FIGURE 2:**
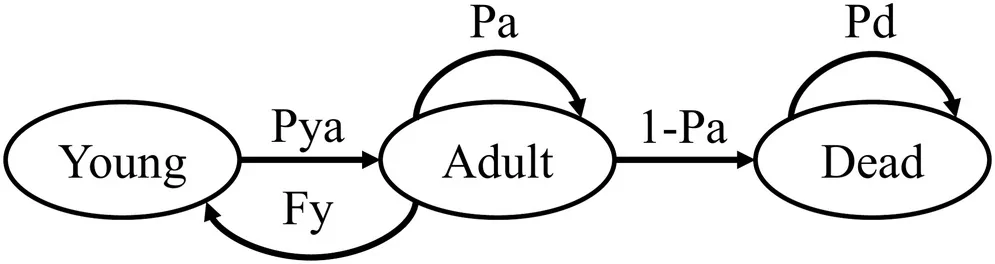
The modelled life cycle diagram and the transition parameters. Pya is the rate of transition from young to adult, Pa and Pd are the survival of the adults and dead shells, respectively, while Fy is the fecundity, i.e., the number of young produced by an adult.

We implemented and analysed the model in R. Stable stage distributions and asymptotic population growth rates (λ) were calculated using the ‘stable.stage’ and ‘lambda’ functions from the ‘popbio’ package (Stubben and Milligan 2007). Overestimation of adult abundance due to inclusion of dead shells was quantified as (p_adult_ + p_dead_)/p_adult_, where p_*i*_ is the proportion of stage *i* in the stable stage distribution.

To investigate the effect of population dynamics on overrepresentation, we systematically varied the parameters: Pya (0.1–0.9, step 0.2), Fy (10, 20, 50) and both adult survival (Pa) and dead shell persistence (Pd) (0–0.9, step 0.1). For each parameter combination, we calculated the overestimation factor. To better understand the effect of life histories, we also constructed two sets of variables representing the slow and fast pace of life (Vasilieva 2022). The ‘fast’ model was calibrated to a higher young mortality (Pya: 0.1) and higher fecundity (Fy: 50); meanwhile, the ‘slow’ model was calibrated to a higher young‐to‐adult transition (Pya: 0.9) and lower fecundity (Fy: 10).

## 3 Results

### 3.1 The Effects of Conservation Management: Comparing the Live and Combined Data Sets

The analysis of structural changes in the community revealed markedly different results between the two data types (Figure 3).

**FIGURE 3:**
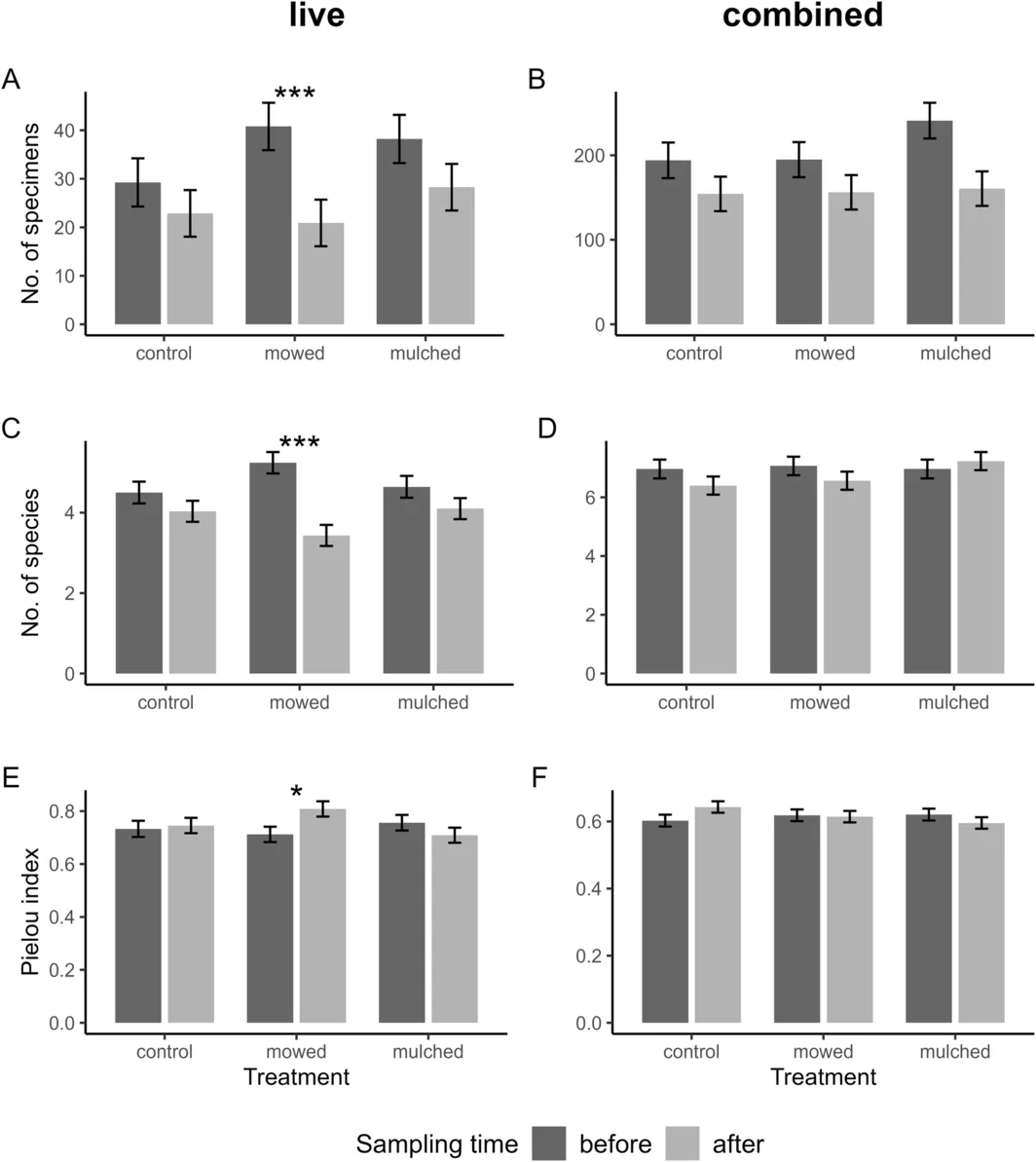
The impact of treatment based on live (left) and combined data (right). The impact of treatment on the number of individuals (top), the number of species (middle) and the evenness (bottom). Vertical error bars indicate standard errors of the estimates derived by the emmeans function from the fitted GLMM models (***p < 0.001; *p < 0.05; where no ‘*'s are given, the difference is not significant, i.e., *p* > 0.05). The effect of management on the whole area, expressed as the interaction between treatment and sampling time, was evaluated using Likelihood Ratio Tests (LRTs). In the live dataset, the interaction term significantly affected the number of specimens (*χ* ^2^_2_ = 6.993, *p* = 0.030), the number of species (*χ* ^2^_2_ = 11.318, *p* = 0.004), and Pielou's evenness index (*χ* ^2^_2_ = 6.275, *p* = 0.043). In contrast, no significant interaction effects were detected in the combined dataset for number of specimens (*χ* ^2^_2_ = 0.721, *p* = 0.698), number of species (*χ* ^2^_2_ = 4.154, *p* = 0.125), or evenness (*χ* ^2^_2_ = 3.786, *p* = 0.151).

Calculations based on the live data revealed a significant effect of mowing: species richness and abundance decreased between sampling occasions in mowed plots, whereas diversity, as indicated by the Pielou index, increased (Farkas et al. 2025). In contrast, the combined data did not indicate a significant effect of any treatment on these variables.

### 3.2 Differences in Abundance of Species

During the species‐level review of the differences between the two data types, we observed substantial interspecific variation in the degree of overrepresentation (combined abundance/live abundance). The maximum value was an almost twelvefold difference. The minimum is one (the lower limit of this variable, which means that there were no empty shells, only living individuals), and we can see a smooth transition between these two extremes (Figure 4). The overrepresentation for most species is between 2 and 6.

**FIGURE 4:**
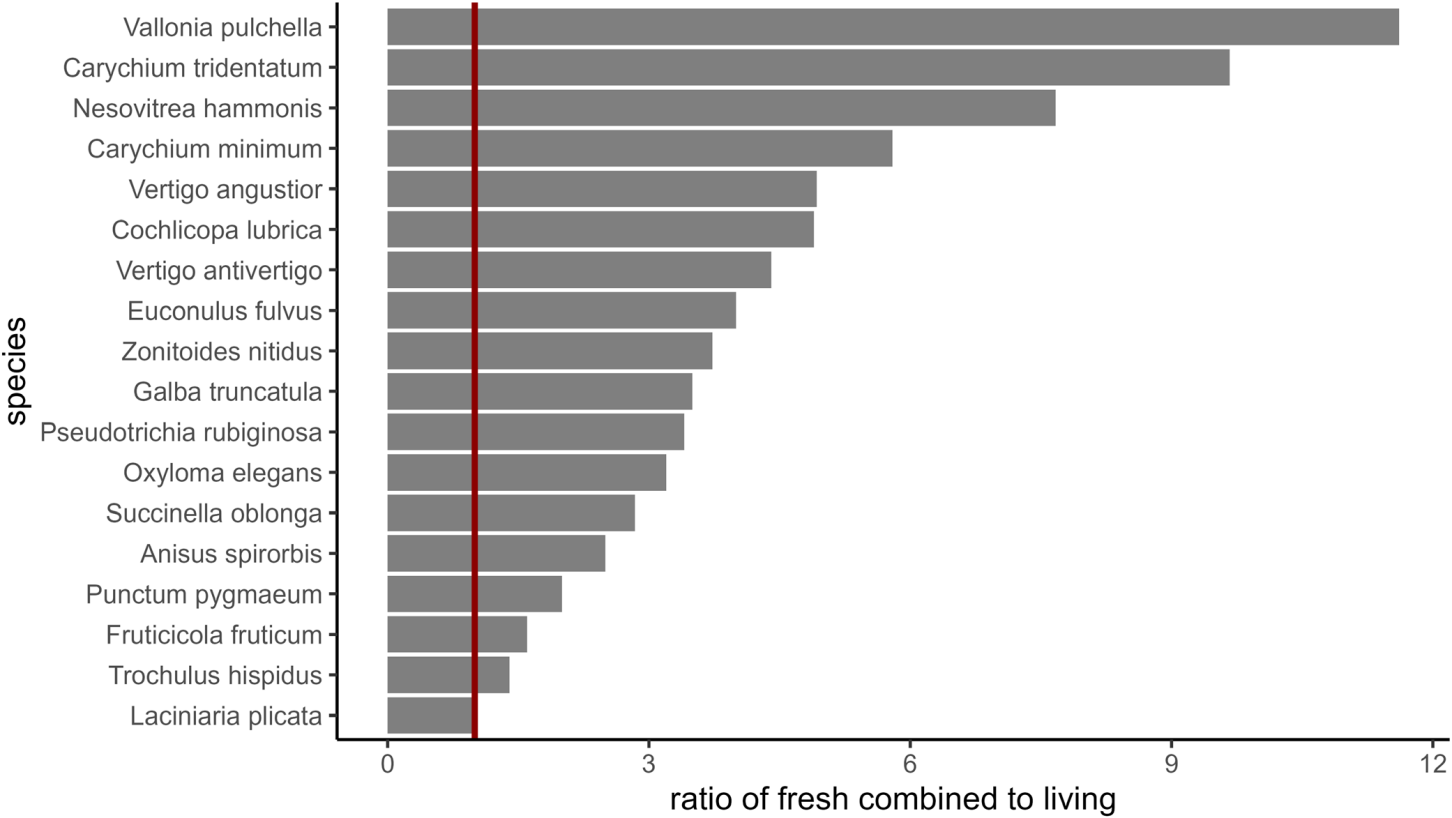
Overrepresentation values by species: The ratio of combined abundance (number of fresh empty shells and living individuals) to living abundance (number of living individuals). The red line marks the reference value of 1. In each bar, the lengths of the segments on either side of that line indicate the relative contribution of live individuals (left) and fresh empty shells (right) to the combined abundance. Size (mm^2^) of the species in order of their overrepresentation values are: *T. hispidus* —55.18, *P. pygmaeum* —2.40, *A. spirorbis*—11.86, *S. oblonga* —19.19, *O. elegans* —37.55, *P. rubiginosa* —31.48, *G. truncatula* —9.32, *Z. nitidus* —24.90, *E. fulvus* —7.45, *V. antivertigo*—2.53, *C. lubrica* —13.63, *V. angustior*—1.69, *C. minimum* —1.40, *N. hammonis*—11.13, *C. tridentatum* —1.39, *V. pulchella* —4.36. Size of *L. plicata* and *F. fruticum* are missing because not enough specimens were available to measure.

### 3.3 Relationships Between Resistance and Shells Characteristics

Our results (Figure 5) indicate a strong positive relationship between shell size and both physical (*t* = 6.74, df = 9, *p* < 0.001) and chemical resistance (*χ* ^2^ = 23.50, df = 1, *p* < 0.001). Regarding shell shape, no significant relationship was found with physical resistance (*F* = 4.02, df = 1, 8, *p* = 0.080), and its relationship with chemical resistance was non‐linear. Specifically, shells exhibited lower chemical resistance when they were rounded or elongated, as indicated by a weak but significant quadratic term (*χ* ^2^ = 6.07, df = 2, *p* = 0.048).

**FIGURE 5:**
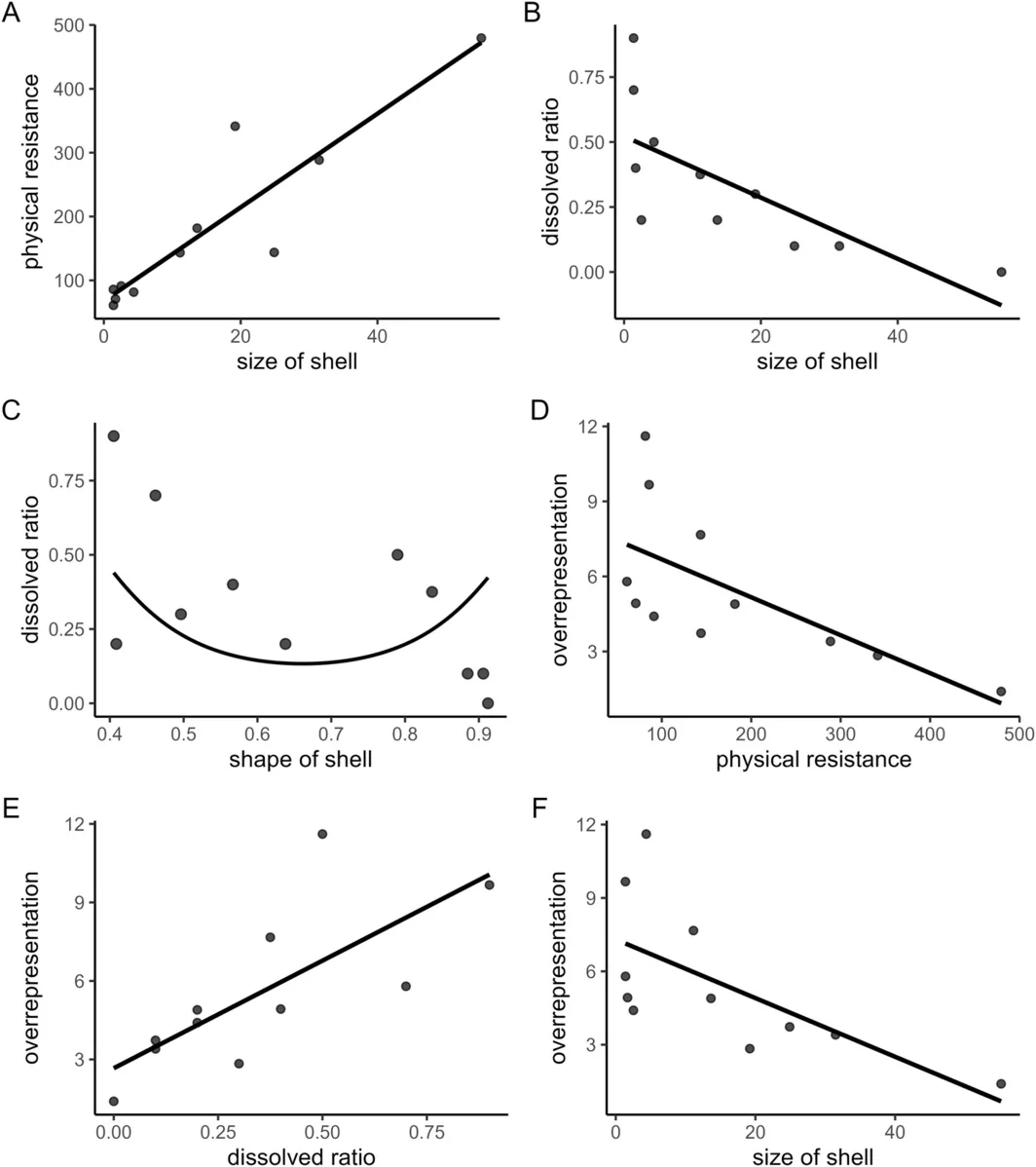
Significant relationships between physical resistance, dissolved ratio (the opposite of chemical resistance, *n*_dissolved/*n*), size and shape of the shells and overrepresentation (combined abundance/live abundance). For statistical details, see the main text.

### 3.4 Relationships With Overrepresentation

We found that shell size and both resistance values have a significant relationship with overrepresentation. Surprisingly, species with more resistant shells show lower levels of overrepresentation (physical res.: *χ* ^2^ = 8.18, df = 1, *p* = 0.004; inverse chemical res.: *z* = −3.25, df = 9, *p* = 0.001). On the other hand, smaller shell sizes are associated with a greater overrepresentation (*χ* ^2^ = 14.19, df = 1, *p* < 0.001).

### 3.5 Effect of Life Cycle Differences

The multistage population model confirmed our expectation that adult survival (Pa) and dead shell persistence (Pd) are the primary drivers of adult overrepresentation. Our simulations indicated that the degree of overrepresentation, quantified as (p_adult_ + p_dead_)/p_adult_, is positively influenced by the persistence of the dead shells and negatively by adult survival.

Specifically, higher values of dead shell persistence (Pd), lower values of adult survival (Pa), and fast pace consistently led to a greater overrepresentation of dead shells in the stable stage population (Figure 6).

**FIGURE 6:**
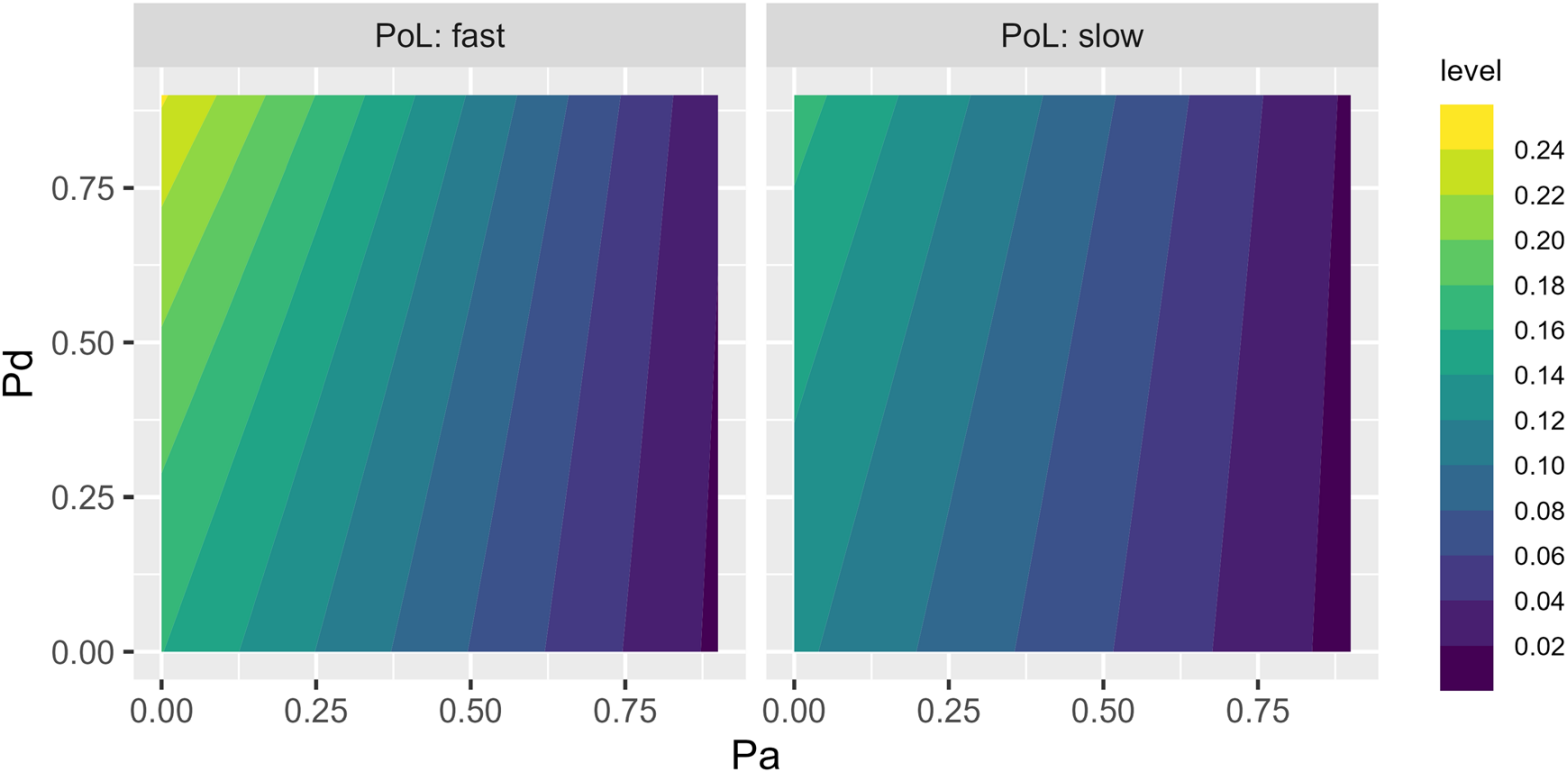
Two groups of Leslie matrix models, calibrated to two different pace of life strategies. The level of overrepresentation (given as log_10_((p_adult_ + p_dead_)/p_adult_)) is visualised over a range of two parameters: Pd is the probability of fresh empty shells surviving instead of turning into old empty shells by destruction, and Pa is the probability of adult survival. The first model represents species with a fast pace of life: Low young‐to‐adult transition (Pya: 0.1) and higher fecundity (Fy: 50); meanwhile, the second one represents species with a slower pace of life: High young‐to‐adult transition (Pya: 0.9) and lower fecundity (Fy: 10).

## 4 Discussion

Animal remains can provide valuable information on species occurrence and abundance that is often difficult to obtain from surveys of living individuals alone. However, meaningful interpretation of these materials requires an understanding of both the biology of the studied taxon and the processes that modify remains after deposition (Henderson 2021).

Here we compared the two primary data types used for the assessment of terrestrial gastropod communities (live data type: only live snails included, combined data type: live individuals + fresh empty shells included). Using the data obtained from the short‐term grassland management study of Farkas et al. (2025), we found that the negative impact of mowing on gastropod abundance and species richness reported by using live data (Farkas et al. 2025) was undetectable when using combined data.

Exploring the underlying causes, we found substantial interspecific variation in the ratio of combined abundance to live abundance. This suggests that the combined data type may provide biased information regarding the instantaneous state of the community (acting like a ‘distorted mirror’: while all elements are visible, their proportions are altered).

One possible explanation for this was that resistant shells degrade more slowly, thereby remaining in the ‘freshly dead’ category for a longer duration. Under this assumption, species with more resistant shells would exhibit higher overrepresentation values. Another possibility was that species produce new empty shells at different rates due to variations in their life history. We investigated both possibilities.

By integrating our laboratory shell‐resistance data, shell size parameters, and the degree of overrepresentation, we identified a partially unexpected relationship. As anticipated, species with smaller shell sizes exhibited lower resistance, with their shells being more prone to fracturing and acid dissolution. Nevertheless, their empty shells were present in relatively high proportions, resulting in higher overrepresentation values compared to larger species. Thus, interspecific differences in overrepresentation were not explained by shell resistance as expected. However, these findings highlighted the significance of shell size. This is consistent with the findings of Sólymos et al. (2009), namely, the proportion of empty shells (relative to live individuals) was higher in snail communities with a higher proportion of smaller species.

In the literature (e.g., Künkel 1928; Frömmig 1954; Uminski and Focht 1979; Baur 1989; Myzyk 2011; Maltz and Sulikowska‐Drozd 2012; Welter‐Schultes 2012), we found that smaller species more frequently exhibit shorter life cycles, characterised by multiple generations per year, faster maturation and shorter lifespans. Incorporating these traits into a Leslie matrix‐based modelling exercise, we recovered our earlier observation: smaller size can be associated with greater overrepresentation. This means that life‐history traits governing the accumulation of dead shells are key factors responsible for the discrepancies observed between live and combined datasets. In other words, in the community we studied, species with smaller, therefore more fragile shells usually have a faster pace of life. These species produce adult empty shells faster, at such a high rate that despite the fact of their low resistance (and hence probably faster destruction), the ratio of adult fresh empty shells to adult living individuals remains high.

Sólymos et al. (2009) also put forward an alternative hypothesis, namely, different rates of shell accumulation (i.e., overrepresentation) may be linked to the different microhabitat preferences of the species. This explanation probably does not explain the differences in overrepresentation we found because we sampled snails from a rather uniform habitat where microhabitat differences might be small.

It must be emphasised that our study is based on data from two adjacent sites within a single habitat type. This naturally limits the generalisability of our conclusions; however, our primary finding (that the choice of data type strongly influenced the assessment of changes within the studied community) warrants serious consideration of the issue. We recommend including additional habitat types and communities in future similar investigations.

To summarise, the observed interspecific differences in abundance ratios between the two data types may be driven by divergent life‐history strategies among species. The discrepancy between the two data types is substantial; therefore, the selection of the appropriate data type (in‐ or excluding fresh empty shells) must be a deliberate step in research design for community‐level studies of terrestrial gastropods. For the detection of short‐term temporal changes, we recommend prioritising the use of live data. We consider it essential that the choice of methodology and its justification be explicitly stated in scientific publications. Beyond facilitating reproducibility, this is fundamental for the accurate interpretation of research findings.

## Author Contributions

**Nóra Kriston‐Kőműves:** conceptualization (equal), data curation (equal), formal analysis (lead), investigation (lead), methodology (equal), visualization (equal), writing – original draft (lead). **Roland Farkas:** conceptualization (equal), data curation (equal), methodology (equal), resources (lead), writing – original draft (supporting), writing – review and editing (supporting). **Zoltán Barta:** conceptualization (equal), funding acquisition (lead), methodology (equal), project administration (lead), software (lead), visualization (equal), writing – original draft (supporting), writing – review and editing (lead).

## Funding

This work was supported by Nemzeti Kutatási, Fejlesztési és Innovaciós Alap, EKÖP‐25‐2‐DE‐161, K138503.

## Conflicts of Interest

The authors declare no conflicts of interest.

## Data Availability

The data that support the findings of this study are openly available in Nóra, Kriston‐Kőműves; Roland, Farkas; Zoltán, Barta, 2026, ‘Snail inventories for short‐term impacts: Methodological differences and their underlying causes’, https://doi.org/10.48428/ADATTAR/A8OZ2B, University of Debrecen, V1.
